# Sex, gut microbiome, and cardiovascular disease risk

**DOI:** 10.1186/s13293-019-0240-z

**Published:** 2019-06-10

**Authors:** Alexander C. Razavi, Kaitlin S. Potts, Tanika N. Kelly, Lydia A. Bazzano

**Affiliations:** 1 Department of Medicine, Tulane University School of Medicine, New Orleans, LA USA; 2 Department of Epidemiology, Tulane University School of Public Health and Tropical Medicine, 1440 Canal Street, Suite 2000, New Orleans, LA 70112 USA

**Keywords:** Gut microbiome, Cardiovascular diseases, Sex difference, Obesity, Lipids, Insulin, Blood pressure, TMAO

## Abstract

Key differences exist between men and women in the determinants and manifestations of cardiovascular and cardiometabolic diseases. Recently, gut microbiome-host relations have been implicated in cardiovascular disease and associated metabolic conditions; therefore, gut microbiota may be key mediators or modulators driving the observed sexual dimorphism in disease onset and progression. While current evidence regarding pure physiological sex differences in gut microbiome composition is modest, robust research suggests that gut microbiome-dependent metabolites may interact with important biological pathways under sex hormone control, including toll-like receptor and flavin monooxygenase signaling. Here, we review key sex differences in gut microbiome interactions with four primary determinants of cardiovascular disease, impaired glucose regulation, dyslipidemia, hypertension, and obesity. Through this process, we propose important sex differences in downstream metabolic pathways that may be at the interface of the gut microbiome and cardiovascular disease.

## Background

It is increasingly appreciated that the human gut microbiome, a network that includes over 100 trillion bacteria, and its changes over the lifespan are involved in the pathophysiology of cardiovascular disease (CVD) [1–3]. For example, gut microbial-dependent metabolites including, short-chain fatty acids (SCFAs) and trimethylamine *N*-oxide (TMAO), may modify CVD determinants through G protein-coupled receptors (GPCR) that modulate blood pressure [4] or through inhibition of high density lipoprotein (HDL)-coordinated reverse cholesterol transport [2], respectively. The extent to whether such microbe-host physiology exhibits sexual dimorphism in the setting of CVD remains largely unexplored, as these dynamic relationships have not been clearly defined or systematically reviewed across both men and women.

This review focuses on the biological pathways underlying sex differences in CVD, particularly involving novel relationships between the gut microbiome and CVD risk factors. We will first review sex differences regarding four primary determinants of disease including blood pressure, lipid metabolism, glucose metabolism, and body weight. We will then introduce the gut microbiome, highlighting its intricate relationship with the human diet, and discuss the downstream microbiome-dependent metabolites and pathways influencing CVD. Through this process, we will assess the current evidence regarding the relationships of the gut microbiome with blood pressure, serum lipid, and glycemic profiles, as well as body weight, and the potential influence of sexual dimorphism in these gut microbiome-host relations.

## Sex differences in CVD and CVD risk factors

CVD is responsible for the greatest proportion of deaths in both men and women, with CVD mortality rates approximately 32% and 35%, respectively [5, 6]. While age-adjusted CVD mortality rates are higher in men compared to premenopausal women [6, 7], one third of women in the USA are affected by CVD, and nearly 50% of women in Western countries will die from coronary heart disease or stroke [8]. Furthermore, whereas clinical and public health CVD efforts must continue to target both sexes equally, key differences in the epidemiology and pathophysiology of risk factors have been identified in men and women. These corresponding differences underline the need to examine the role of sex in the development and progression of CVD and its respective upstream disease risk factors.

A large body of evidence has demonstrated sex differences in CVD risk over the past several decades. Impaired glucose regulation, dyslipidemia, hypertension, and obesity are among the most important CVD risk factors in the general population. Table 1 highlights fundamental biological sex differences in these four risk factors as well as the evidence and potential underlying mechanisms that may mediate such observations.

**Table 1:** Sexual dimorphism in four main cardiovascular disease risk factors

CVD risk factor	Men	Women	Evidence/potential mechanisms
Impaired glucose regulation^A^	**↑** Incidence of impaired fasting glucose **↑** Incidence of diabetes at earlier ages **↓** Insulin sensitivity	**↑** Incidence of impaired glucose tolerance **↓** Incidence of diabetes **↑** Insulin sensitivity	Estrogens may confer a protective effect on insulin-glucose homeostasis [172–178]: - Reduction in inflammation, reactive oxygen species, hepatic glucose production, and central and visceral adiposity. - Improves glucose uptake by skeletal muscle via activation of PPAR-γ. Testosterone appears to exhibit a U-shaped association with insulin resistance [179–183]: - Excess testosterone in both sexes is associated with dysglycemia and inhibits myocyte, adipocyte insulin in women. - Testosterone associates with reduced visceral and central adiposity, as well as decreased waist-to-hip ratio in men.
Dyslipidemia^B^	**↓** HDL-C **↑** LDL-C **↑** VLDL **↑** Total plasma TG **↑** FFA oxidation at rest	**↑** HDL-C **↓** LDL-C **↓** VLDL **↓** Total plasma TG **↑** FFA storage at rest	Sexual dimorphism is observed in lipid profiles of premenopausal women compared to men [184–187]
Hypertension^C^	Younger ages [118, 188–190] ↑ Systolic BP ↑ Incident hypertension ↓ Salt sensitivity Older ages ↓ Incident hypertension All ages ↑ Diastolic BP ↓ Survival with hypertension	Younger ages ↓ Systolic BP ↓ Incident hypertension ↑ Salt sensitivity Older ages (postmenopausal) ↑ Incident hypertension All ages ↓ Diastolic BP ↑ Survival with hypertension	Endogenous estrogen has a BP lowering effect [118, 191–194]. - Possible mechanisms include RAAS and endothelin system, oxidative stress, nitric oxide production, and salt sensitivity. Androgens (testosterone) have pro-hypertensive properties. - Possible mechanisms include blunting of the pressure-natriuresis relationship, RAAS, and oxidative stress.
Obesity^D^	↓ Obesity [195] ↑ Lean tissue; ↓ Total fat ↑ Visceral adipose tissue	↑ Obesity [195] ↓ Lean tissue; ↑ Total fat ↑ Subcutaneous adipose tissue	Estrogen and androgens impact energy utilization, storage, and fat distribution [196–199].

^A^Broad category of prediabetic syndromes, including impaired fasting glucose (WHO criteria, > 110 mg/dL; ADA criteria, > 100 mg/dL) as well as impaired glucose tolerance, a condition in which a given concentration of insulin, endogenous or exogenous, is accompanied by an inadequate glucose response

^B^An elevation in circulating total cholesterol, low-density lipoprotein, high-density lipoprotein, and/or triglycerides

^C^Defined by ACC/AHA 2017 guidelines: systolic blood pressure ≥ 130 mmHg or diastolic blood pressure ≥  80 mmHg

^D^Defined by a body mass index ≥ 30 kg/m^2^

*BP* blood pressure, *FFA* free fatty acids, *HDL-C* high-density lipoprotein cholesterol, *LDL-C* low-density lipoprotein cholesterol, *PPAR-γ* peroxisome proliferator-activator gamma, *RAAS* renin-angiotensin-aldosterone system, *VLDL-C* very low-density lipoprotein cholesterol

## Introduction to the microbiome

The human microbiota represents the collection of microorganisms that live in and on the human body, including the gastrointestinal tract, urogenital system, and skin. The human microbiome, precisely, refers to the genomes of such microorganisms, including bacteria, fungi, archae, protists, and viruses [9]. While all five latter microorganisms are found in the human gut, bacteria are the most prevalent and well-studied, and relationships of the virome, mycobiota, and archae with human health remain largely unexplored. Microbial cells outnumber host cells in the human body, and the gut microbiome plays a critical role in host metabolism, physiology, and susceptibility to and risk of disease, particularly CVD [10]. Our gut microbiota, predominantly bacteria, helps absorb and metabolize food constituents, producing biologically active microbial metabolites that proceed through the portal system, entering systemic circulation to influence human physiology.

## Diet and the gut microbiome

The gut microbiome serves as a filter for perhaps the most common human environmental exposure, diet. Our diets are one the most important modulators of microbiota composition and its respective metabolites, notably TMAO and SCFA [11]. While sex differences were not explored in the analysis, one study comparing children adherent to a rural diet in Burkina Faso (vegetarian, high fiber, low fat) versus a modernized western diet in Europe (animal protein, low fiber, high fat) found that rural children had significant increases in *Bacteroidetes* phyla as well as *Prevotella* and *Xylanibacter* genera and a reduction in the *Firmicutes* bacterial phylum [12]. Together, this microbial composition also led to a significant increased production of the three most prevalent SCFA, acetate, propionate, and butyrate. Thus, diets high in fiber and plant protein as well as low in saturated fat may lead to increased microbial richness and more abundant production of SCFA [12]. SCFAs are fermentation by-products of carbohydrates and proteins that help maintain the integrity of the intestinal brush border but may also reduce CVD risk through reductions in systolic blood pressure and serum cholesterol, as well as through improved insulin sensitivity [13, 14]. Similar to SCFA, gut microbiota-dependent metabolite, TMAO, is intricately associated with dietary intakes. TMAO has been causally associated with atherosclerosis, and this metabolite derives from foods rich in choline, phosphatidylcholine, and carnitine [11]. The latter three dietary metabolites are predominantly found in animal-based foods, including eggs, red meat, and dairy, and studies in vegetarians and vegans have confirmed that individuals adherent to plant-based diets produce less TMAO compared omnivorous to subjects [15] (Table 2). Mechanistically, dietary foods that contain TMAO metabolite substrates are converted by gut microbial enzymes to trimethylamine, which is subsequently oxidized by hepatic flavin monooxygenase 3 (FMO3) to yield TMAO [3].

**Table 2:** Sexual dimorphism in four main cardiovascular disease-related metabolites

Metabolite	Men	Women	Cardiovascular disease risk
Branched-chain amino acids	**↑** Serum branched-chain amino acids **↓** Branched-chain 2-oxoacid dehydrogenase	**↓** Serum branched-chain amino acids **↑** Branched-chain 2-oxo acid dehydrogenase	Increased risk of insulin resistance and type II diabetes in men compared to women - Possible mechanisms include female sex hormone regulation of branched-chain 2-oxoacid dehydrogenase and enrichment of the gut microbial *Bacteroides*-*Prevotella* group in men [30, 72].
Short-chain fatty acids	**↓** Short-chain fatty acids **↓** Dietary fiber intake **↓** PPAR-γ	**↑** Short-chain fatty acids **↑** Dietary fiber intake **↑** PPAR-γ	Increased susceptibility to dyslipidemia in men compared to women - Possible mechanisms include 17β-estradiol-mediated increase in PPAR-γ receptor expression and decreased dietary fiber intake in men [20, 82].
Trimethylamine *N*-oxide	**↓** TLR expression **↓** FMO3 expression **↓** Secondary bile acids	**↑** TLR expression **↑** FMO3 expression **↑** Secondary bile acids	Greater thrombotic risk in women compared to men - Possible mechanism: increased TLR and trimethylamine *N*-oxide activation of platelets. [54, 55]. Accelerated trimethylamine *N*-oxide production in women compared to men - Possible mechanisms: gonadal hormone regulation of hepatic FMO3 expression and increased secondary bile acid activation of Farnesoid X receptor [43, 87].
Lipopolysaccharide	**↓** TLR4 expression **↓** TLR2 signaling	**↑** TLR4 expression **↑** TLR2 signaling	Estrogens, progesterone, and testosterone regulate LPS-mediated signaling through TLR4 [62–64].

*FMO3* flavin monooxygenase-3, *PPAR-γ* peroxisome proliferator activating receptor gamma, *TLR* toll-like receptor

Differences in dietary intake between men and women may thus be an important source of sexual dimorphism in CVD risk. While not all reports have observed sex differences in diet [16, 17], several studies have reported that men consume fewer high-fiber foods, including fruits and vegetables, and have higher dietary intakes of fat and salt compared to women in both childhood and adulthood [18–20]. Likewise, consistent associations have been reported between specific foods and gender, with red meat and alcohol associated with masculinity, whereas femininity has been correlated with fish, fruits, and vegetables [21]. Therefore, differences in dietary intake in men and women, perhaps stemming from societal and behavioral factors, may be important to consider when assessing the role of the gut microbiome in sexual dimorphism in CVD and its associated risk factors.

## Sex differences in the microbiome

Sexual dimorphism in the gut microbiome may be influenced by genotype, diet, age, ethnicity, geographic location, and/or the health status of the host [22]. Characterizing gut microbiome profiles through bacterial phyla [23, 24] demonstrates high proportions of *Bacteroidetes* and *Firmicutes* phyla in healthy adults, while *Proteobacteria*, *Actinobacteria*, *Fusobacteria*, and *Verrucomicrobia* are phyla less represented [25–27]. Evidence from studies suggests that women may harbor a higher ratio of *Firmicutes*/*Bacteroidetes* (F/B) in comparison to men [28–30]. The F/B ratio, increasing in magnitude from birth to adulthood [31], is used in microbiome studies as it is an important measure of human microbiota composition and appears to be a key component in biological aging and obesity [32]. Additionally, *Firmicutes* and *Bacteroidetes* are the two most common bacterial phyla in the human microbiome; therefore, perturbations in the proportional composition of these two taxonomical groups may provide insight into host health status. *Bacteroidetes* are the most prevalent phylum of gram-negative bacteria occupying the human gastrointestinal tract and are considered to be largely beneficial due to their functional capabilities of polysaccharide degradation and regulation of calorie absorption [33]. With respect to *Firmicutes*, most gut bacteria representing this phylum are gram-positive and are capable of producing several SCFAs, which may contribute to a protective CVD phenotype through improved blood pressure control and glucose homeostasis [13]. The F/B ratio is heavily influenced by BMI [34] and thus may play a significant role in regulation of adiposity. Among those with a BMI greater than 33, a significantly lower F/B ratio has been seen in men compared to women, while the opposite holds true in those with a BMI less than 33 as well as in postmenopausal women [35]. Adjusting for BMI, higher proportions of *Firmicutes* have been found in women compared to men. With respect to other less represented gut microbiome phyla, higher numbers of *Proteobacteria*, *Veillonella*, and *Blautia* have been reported in women in comparison with men [29, 35, 36]. The F/B ratio has been used as an indicator of gut dysbiosis, with a higher F/B ratio representing a more dysbiotic microbiome.

In addition to compositional differences, sex-specific heterogeneity may exist in microbiome responses to external stimuli, including diet. In one study of Japanese individuals between 18 and 23 years of age, sex was found to modify the relationship between yogurt consumption and gut microbiome composition. Regular yogurt consumption was associated with a higher proportion of *Lactobacillus casei* in women yet was negatively associated with microbiome concentrations of *Lactobacillus sakei*, *Enterobacteriaceae*, and *Staphylococcus* in men [37]. *Lactobacilli* are the most common species found in probiotic preparations [38] currently being investigated for benefit in several gastrointestinal diseases, such as ulcerative colitis [39] and irritable bowel syndrome [40]. While findings from the noted research may suggest that sex biologically modifies the relationship between diet and the gut microbiome, investigators in this study did not control for important covariates including BMI or baseline diet.

Very few studies have specifically explored gut microbiome differences between men and women as a primary research question, as much of the current evidence stems from sensitivity and post hoc analyses. Additionally, while a significant body of evidence demonstrates that early infant life and age are key determinants of gut microbial composition, no prospective longitudinal studies tracking potential sex differences in the gut microbiome across the lifespan have been conducted.

## The microbiome and CVD risk factors: role of sex differences

**Fig. 1:**
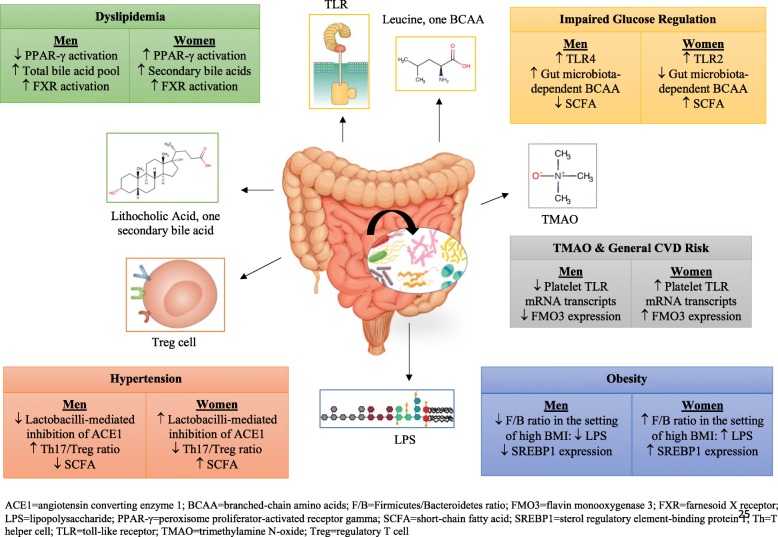
Proposed mechanisms by which gut microbiota mediate sex differences in cardiovascular disease risk

Bidirectionality is an important consideration when describing gut microbiome changes in relation to respective CVD risk factors. Dyslipidemia, dysglycemia, hypertension, and obesity may all induce or themselves be modified by gut microbiome changes [41] (Fig. 1). In spite of the prematurity of the scientific discipline and the need for longitudinal studies to establish temporality, there is a considerable amount of evidence to parse regarding sex-specific differences underlying the relationship of the gut microbiome and traditional CVD risk factors. The focus here is to highlight aspects of microbiome-CVD risk factor relationships that may be a result of or contribute to observed sex differences in disease.

Some of the evidence implicating microbiota with CVD risk factors and identifying sex differences in these relationships comes from experimental studies in mice that have used various biological mouse models. For example, apolipoprotein E-deficient mice are atherosclerosis-prone and have been used to assess the role of microbiota in atherosclerotic processes [42]. Ovariectomy and castration in female and male mice, respectively, allow for the study of hormonal influences on physiology and susceptibility to disease [43]. Germ-free mice are commonly used in microbiome-related research as they are raised in conditions that render them completely free of all (detectable) microorganisms. This offers the possibility of studying effects in the total absence of microbes (germ-free) and in the presence of known microbes (gnotobiotic) once introduced to the germ-free mice, for example after fecal transplantation. This model allows for studying the temporal and near direct effects of the gut microbiome on phenotypes, as gut microbiota are transferred from donor mice with a particular disease phenotype to recipient germ-free mice [44]. An alternative method to using germ-free mice is antibiotic treatment to depress resident microbes prior to introducing specific microbes for study [45]. Knockout mice have also been used to assess the impact of a loss of certain genes on the relationship between the microbiome and CVD risk factors [46].

## Microbiome and markers of cardiovascular disease risk

Direct evidence for the involvement of the gut microbiome in the etiology of CVD comes from TMAO [47], a gut microbiome-dependent plasma metabolite that has been associated with increased CVD risk and events in several human and animal studies [47, 48]. TMAO is a prevalent metabolite in animals and humans, serving as an osmolyte particularly in the kidney, and high plasma concentrations of TMAO may suggest both underlying CVD and/or renal disease [49]. Of the metabolite’s many functions, TMAO modulates cholesterol metabolism in the liver, intestines, and arterial walls. When TMAO is present in systemic circulation, there is increased accumulation and decreased removal of cholesterol from peripheral endothelial cells lining arterial walls [50]. Circulating TMAO levels trigger increases in pro-inflammatory cytokine expression, leukocyte recruitment, and adhesion molecules, inducing vascular inflammation [51]. Wang et al. were able to prevent atherosclerosis in apolipoprotein E-deficient mice by reducing plasma TMAO levels [52], and TMAO levels may predict adverse cardiovascular events [53]. Heart failure patients have increased levels of TMAO compared with age- and sex-matched controls, and elevated TMAO is also associated with shorter survival in heart failure [48]. The cardiovascular risks of varying TMAO plasma levels were transferrable by gut microbiota transplantation in antibiotic-treated mice [45].

Overall, men may harbor protective physiological mechanisms with respect to endogenous TMAO production. Sexual dimorphism in the TMAO pathway may be attributable to diet, genetics, and hormones, as well as renal and immuno-physiologic factors. TMAO increases both platelet reactivity and thrombotic risk [54]. Sex differences in TMAO-induced platelet activation may be mediated by toll-like receptors (TLR); women contain more TLR mRNA transcripts compared to men [55], potentially making them more vulnerable to the adverse cardiovascular effects of TMAO. FMO3 catalyzes the rate-limiting step in TMAO production. Sex differences in hepatic FMO3 expression have been reported, with women expressing higher levels of this TMAO-producing enzyme compared to men [43]. FMO3 may be under hormonal regulation, as castrated male mice experience an over 100- and 7-fold rise in FMO3 mRNA and TMAO levels, respectively [43]. Estrogen, although to a smaller magnitude than androgens, also seems to influence FMO3 expression; estrogen supplementation in ovariectomized mice increases FMO3 expression. In total, these results suggest that androgens are the primary drivers of sex differences in hepatic FMO3 expression, with estrogens complementarily widening this difference although by a much smaller magnitude. Androgen-dependent reduction in FMO3 expression is thus a potential protective factor in the setting of atherogenic CVD. Farnesoid X receptor (FXR), which plays a role in bile acid receptor signaling [56, 57], may also regulate TMAO production via FMO3. Similar rises in plasma TMAO across both sexes are noted upon synthetic FXR activation in mice [43]. Though not previously reported, we believe that FXR-induced TMAO production may partially explain the observed potential deleterious effects of FXR on CVD risk, and that microbiome-derived secondary bile acids are a possible mediator of sexual dimorphism in this pathway. Women reportedly contain higher circulating concentrations of gut microbiome-dependent secondary bile acids compared to men, and this physiological manifestation may accelerate TMAO production, subsequently increasing atherogenic and thrombotic risk.

### Microbiome and impaired glucose regulation

Gut microbiome dysbiosis has been implicated in the pathogenesis of type II diabetes [41]. Individuals with type II diabetes have both functional and compositional gut microbiota differences compared to those without disease [58]. Transfer of fecal microbiota from healthy human hosts to individuals with metabolic syndrome has increased gut microbial diversity and improved insulin sensitivity [59]. Such evidence demonstrates a role for the gut microbiome in the development of glycemic dysregulation and type II diabetes; however, mechanistic pathways and sex-specific pathophysiology remain to be elucidated.

Among the number of mechanisms by which gut dysbiosis could contribute to insulin resistance, one primary means may be through systemic low-grade inflammation [60]. Inflammation can disrupt insulin sensitivity via TLR signaling cascades. Toll-like receptor 2 (TLR2) knockout mice exhibit insulin resistance and glucose intolerance associated with key modifications in intestinal microbiota, including higher proportions of *Bacteroidetes* and *Firmicutes* coupled with a lower proportion of *Proteobacteria* phyla [46]. Insulin resistance associated with absent TLR2 signaling may be attributed to increased serum lipopolysaccharide (LPS) activation of toll-like receptor 4 (TLR4) in the muscle, liver, and adipose tissue [61]. Sex-specific expression and signaling through both TLR2 and TLR4 have been reported [62], particularly through sex hormones. Testosterone decreases TLR4 expression in macrophages [63], and exogenous estrogen treatment in mice increases cell membrane expression of TLR4 [64], while progesterone diminishes LPS-mediated TLR4 signaling [65]. Therefore, although no studies have specifically examined the potential modifying effects of sex on the relationship between the gut microbiome and insulin resistance, sex-specific activation of inflammatory pathways is an important area for future research.

The relationship between the gut microbiome and insulin sensitivity may be modified by the serum metabolome. Serum triglycerides [66], membrane phospholipids [67], and branched-chain amino acids (BCAAs) [68] are associated with insulin resistance and type II diabetes. Gut microbiota are intricately involved in metabolite biochemical pathways, helping synthesize vitamins, SCFAs, and amino acids, but also facilitate bile acid transformation and hydrolysis of non-digestible molecules [69]. In one study of 300 Danish individuals, insulin resistance was characterized by high serum concentrations of BCAAs and high proportions of gut microbiota *Prevotella copri* and *Bacteroides vulgatus* species, which have a high biosynthetic potential for producing BCAAs [70]. Notably, sex differences have been reported in BCAA and related degradation product metabolism, with men exhibiting higher serum metabolome concentrations of BCAA compared to metabolically similar women [71]. These findings are in line with data demonstrating that the *Bacteroides*-*Prevotella* groups are more prevalent in men compared to women [30]. In an animal model, female rats have more pronounced diurnal variation in hepatic branched-chain 2-oxoacid dehydrogenase (BCODH) activity, with over a twofold increase in morning expression of BCODH compared to male rats [72]. BCODH facilitates the catabolism of circulating BCAAs. Female sex hormones may be responsible for BCODH diurnal variation, as gonadectomy inhibits diurnal variation in female but not male rats [72]. Given that higher serum BCAA concentrations confer increased risk for glucose abnormalities [68, 73], female sex hormone regulation of BCODH may confer a protective effect for insulin resistance and type II diabetes.

### Microbiome and lipids

Gut microbes may affect lipid metabolism through several potential mechanisms. One biologic pathway implicated is gut microbial fermentation of non-digestible carbohydrates. Anaerobic bacteria are uniquely capable of digesting complex carbohydrates, or dietary fiber, with one primary product being SCFAs [41]. There is significant heterogeneity with respect to dietary fiber and SCFA production, of which butyrate, propionate, and acetate are the most abundant. In vitro studies suggest that hydrolyzed guar gums lead to the highest gut microbiota-dependent production of butyrate, while pine fiber and arabinogalactan are the major contributors to acetate and propionate production, respectively [74]. Likewise, fermentation of resistant starch, a dietary and functional fiber found in high amounts in specific foods, including banana flour and rolled oats, favors butyrate production [74]. SCFAs may affect CVD risk via a wide variety of mechanisms including lipid and glucose metabolism, as well as blood pressure modulation [75]. For example, evidence suggests that propionate prevents de novo lipogenesis and cholesterogenesis and may also reduce visceral and liver fat [76]. Such physiology may be mediated through propionate’s activation of GPCR43, a receptor expressed in intestinal and adipose tissue, as well as in immune cells [77]. No sex differences were observed in one rodent study involving production of propionate in response to oligofructose-supplemented diets [78]. Butyrate and acetate have higher selectivity for GPCR41 and GPCR43, respectively, and are both metabolized to become incorporated into fatty acids and cholesterol [13]. Acetate, propionate, and butyrate may interact with peroxisome proliferator-activated receptors (PPARs) in liver, heart, and skeletal muscle tissue, increasing mitochondrial biogenesis and fatty acid oxidation that ultimately lowers lipid levels [79]. PPARs occupy a critical role in the regulation of lipid and carbohydrate metabolism, and sex differences have been reported in the stimulation of PPAR gamma [80]. Pioglitazone, a PPAR-gamma agonist, exhibits stronger efficacy in female mice compared to male mice [81]. This finding may be attributed to 17B-estradiol and a downstream increase in PPAR gamma receptor expression [82]. Besides biological sex, differences in dietary habits and/or genetics are also important variables to consider in the setting of SCFA production and lipid metabolism.

In addition to SCFA, secondary bile acids produced from colonic bacteria may regulate hepatic and systemic lipid metabolism via the bile acid receptor FXR [83]. Hepatic lipids as well as systemic total cholesterol and triglycerides are increased in mice with no expression of FXR, while FXR agonism lowers plasma lipid concentrations [56, 57]. The clinical implication of FXR inactivation is the important role it plays in preventing dyslipidemia, but also hepatic steatosis, a disease that has been closely associated with CVD. In particular, sex-specific expression of lipid-related genes, including *Fas*, *Colla1*, *Timp1*, and *Smpd3*, may be FXR-dependent [84]. FXR knockout mice do not display sex-specific expression of lipid- and bile acid-associated genes [85], suggesting that the interaction of microbiota, bile acids, and FXR may be partially responsible for sexual dimorphism in lipid homeostasis.

Though women have smaller bile acid pools paralleled to men [86], women produce higher concentrations of secondary bile acids compared to men [87]; therefore, perhaps, women harbor more gut microbiota that are capable of bile acid transformations. Gut bacterial species in the colon, especially *Clostridum*, *Eubacterium*, *Ruminococcus*, *Coprococcus*, *Dorea*, *Lachnospira*, *Roseburia*, and *Butyrivibrio* [88, 89], remove bile acid hydroxyl, glycine, and/or taurine groups to yield secondary bile acids that then enter the portal circulation. Secondary bile acids may then activate a number of downstream targets, including FXR, having potential mixed effects on CVD risk [90], leading to a decrease in serum triglycerides and an increase in HDL cholesterol. While an important basis for the sex-specific interplay among the gut microbiome, FXR, and bile acids has been identified, further research is required to explain how these factors subsequently modify lipid-related risk of CVD.

In addition to bile acids, cholesterol-derived steroid hormones may hold an important relationship with gut microbiota in the setting of CVD. Male mice have lower gut microbiome diversity compared to female mice in the same environment [44, 91, 92], and this difference is reduced upon the gonadectomy of male mice. Similarly, animal models demonstrate that gut microbiota are vital in supporting regular estrogen cycles, testosterone concentration, and reproductive roles in men and women [91–93]. Gut bacteria may facilitate the reabsorption of conjugated estrogens, as antibiotic administration has been associated with a 60-fold rise in conjugated estrogen excretion in feces [94, 95]. Bacterial beta-glucuronidase is the primary enzyme involved in deconjugating estrogens for reabsorption in the intestines [96], and the genes encoding this protein are primarily found in the *Firmicutes* phylum [97, 98]. Though levels of *Firmicutes* appear to be influenced by body weight, women may harbor higher intestinal *Firmicutes* compared to men irrespective of BMI [35]. These results suggest that gut microbiota may play a role in key steroid hormone changes across the lifespan that underlie CVD risk, for example, the menopausal estrogen decline and consequent proatherogenic shift of lipid profiles in women.

### Microbiome and blood pressure

The gut microbiota has been implicated in hypertension in both animal and human studies [99–102]. High blood pressure is associated with gut microbiota dysbiosis [103], and the hypertensive phenotype is transferable from humans to germ-free mice through gut microbiota via fecal transplantation [104]. Decreased diversity of gut microbiota has been found in prehypertensive and hypertensive patients [103, 104]. Gut-derived SCFAs, prebiotics, and probiotics have all shown potential to decrease both systolic and diastolic blood pressure in humans [105, 106].

Microbiota production of SCFAs appears to play a pivotal role in the relationship between the microbiome and hypertension. Recent evidence suggests that the blood pressure lowering effects of a high-fiber (prebiotic) diet may act through the production of SCFA acetate by increasing acetate-producing bacteria in the gut [105]. Another study utilizing two independent mouse models found that the SCFA propionate reduced hypertension acting through reduced systemic inflammation via T cell regulation, and resulted in decreased aortic atherosclerotic lesions [107]. Although sex differences were not explored in these studies, differential intake of fiber between men and women may contribute to sexual dimorphism in hypertension, mediated by gut microbiota-dependent SCFA.

Regarding particular bacterial strains, *Lactobacilli* seems to be the most beneficial gut bacteria and has been linked to the antihypertensive effect of foods such as blueberries [108], fermented milk [109], and other probiotics. It should be noted that probiotics have a smaller impact on blood pressure reduction than prebiotic fiber-rich diets acting through increased SCFA production, as described above [105]. The blood pressure-lowering mechanism of *Lactobacilli* may be partially through secretion of peptides that inhibit angiotensin-converting enzyme [99, 110], resulting in decreased ability to convert angiotensin I to angiotensin II, a strong vasoconstrictor. Given that women have been found to have higher levels of *Lactobacilli* in the gut [37], this may partly explain observed lower blood pressure in women prior to menopause compared to men. In addition, men show larger increases in blood pressure in response to angiotensin II than women [111, 112], adding to the potential sex-differential blood pressure effects of varying gut microbiota composition.

The microbiome also acts on hypertension through immune response and inflammation. Gut dysbiosis is shown to lead to increased inflammation, and hypertension is associated with gut dysbiosis, with increased F/B ratio and altered SCFA production [113]. As described previously, the mechanism through which the gut-derived SCFA propionate delivers antihypertensive effects is partly explained by anti-inflammatory immune responses [107]. With further study, these immune-related processes may reveal gut microbiome contributions to sex differences in hypertension. Pro-inflammatory T helper (T_H_) 17 cells are released from actions by gut microbiota [114] and help initiate arterial hypertension [115, 116], and hypertensive male rats have been found to have more T_H_17 cells compared to female rats [112]. Additionally, high salt diets can deplete microbiota diversity, particularly the *Lactobacilli* strain as demonstrated in mice and humans by Wilck et al. [117]. This *Lactobacilli* reduction resulted in increased T_H_17 cells [117]. These findings pose the possibility that higher blood pressure salt sensitivity seen in women [118] may be influenced by reduction in *Lactobacilli* under high-salt environments. Since women may have more *Lactobacilli* than men to begin with [37] and men have a higher number of T_H_17 cells [112], the depletion of the protective strain in women may be of greater magnitude and consequence, resulting in a larger relative increase in T_H_17 cells and a corresponding greater blood pressure effect.

More generally, inflammation has been identified as both a cause and a consequence of hypertension [119] and reduced microbiome diversity can lead to low-grade inflammation [120]. Estrogens can reduce inflammation [121–124], and this activity has been linked to sex differences in the gut microbiome of mice [125].

Gut microbiota production of SCFAs [126, 127] impacts renal sensory nerves and blood pressure [128, 129]. SCFAs, including lactate, acetate, butyrate, and propionate, produced by gut microbiota impact vasodilation and vasoconstriction by acting on cell surface receptors GPCR43, GPCR41, and olfactory receptor 78 [99]. Sex differences in renal functions that regulate blood pressure [130, 131] may be derived in part from microbiome variations.

Recent compelling evidence for the role of the gut microbiome in hypertension comes from a study by Menni et al. that found an inverse association between gut microbial diversity and arterial stiffness, as measured via pulse wave velocity, in women [132]. This association was mostly independent of other metabolic syndrome markers. Further evidence is needed to determine if this effect is similar for men or if this could be a factor contributing to sex differences in hypertension.

### Microbiome and obesity

The gut microbiome has been implicated in the etiology of obesity, particularly through energy extraction [133] as well as energy expenditure [99, 134]. At the same time, obesity alters the composition of the gastrointestinal microbiota [34, 35, 135, 136], indicating a bidirectional relationship.

It is known that diet modulates the composition of gut microbiota in humans and other animals [12, 120, 137–142], but these changes are not easily characterized and can vary greatly by individual [143]. The composition of the gut microbiome responds quickly to large alterations in diet, but it is predominantly influenced by long-term dietary habits [143]. Sex-specific diet preferences, including different macronutrient intakes, are likely strong contributors to sex differences in the microbiome that influence obesity and other metabolic risk factors.

Differential diets result in alterations in microbiome composition as evidenced by the lower F/B ratio, with a higher proportion of *Bacteroidetes* phylum, in people consuming more plant-based fibers compared to those consuming a western diet [12, 144]. The F/B ratio has demonstrated sex- and BMI-dependent differences such that women have a higher F/B ratio, indicative of gut dysbiosis, at high BMI (> 33) compared to men [35]. It has also been shown that the F/B ratio is higher, with increased genera in the *Firmicutes* phylum, in overweight and obese subjects [34, 135, 136]. *Firmicutes* are believed to be important in the development of obesity, and weight loss among obese subjects corresponds with a reduction in total *Firmicutes* such that the F/B ratio realigns with that seen in lean patients [133, 136]. The compositional microbiota differences confer the ability of microbiota in obese individuals to extract more calories from food than microbiota from lean subjects by encoding enzymes that break down otherwise indigestible polysaccharides [133] leading to increased release of LPS endotoxins into circulation. These LPS endotoxins in turn influence fat storage and adipose tissue inflammation in the progression towards obesity [145]. Given this mechanism, the increased proportions of *Firmicutes* that women experience in the presence of obesity indicate a possible mechanism for microbiota in the sexual dimorphism of obesity. Further evidence for the role of gut microbiota in energy harvest is seen in late-stage pregnancy where altered microbiota results in higher energy-yielding communities, increasing the capacity for energy harvest from dietary sources [146, 147].

Gut microbiota-produced SCFAs promote storage of triglycerides [148] through the activation of lipogenic liver enzymes including sterol response element binding protein-1 (SREBP-1) [149]. Compared to men, women may express higher levels of SREBP-1 [150], presenting a possible route for increased lipid storage and increased risk of obesity in women via a microbiota involved pathway. SCFAs also act on obesity development via suppression of the fasting-induced adipocyte factor (FIAF)/angiopoietin-like protein, an important inhibitor of lipoprotein lipase (LPL), as demonstrated in mice gut microbiota [149, 151]. The resulting increased LPL corresponds to a microbiota-mediated increase in fat storage [149] that may be part of the sex difference in body composition and obesity.

In addition to energy homeostasis, the microbiome impacts chronic low-grade inflammation through a variety of mechanisms including the expression of GPR41 and GPR43 activated by gut-produced SCFAs [152] and increases in endocannabinoid system tone [153, 154]. Some mice studies have implicated GPR41 and GPR43 in the chronic inflammatory states of obesity, but the evidence is conflicting [77]. Sex-differential response to GPR41, which is also involved in regulation of energy homeostasis [155], may be an important microbiota-originating mechanism for sexual dimorphism in body weight. Decreased energy expenditure and increased body fat mass were reported in male but not female GPR41 knockout mice compared to their wild littermates [156].

Despite these findings, the role of SCFAs in obesity is still unclear as evinced by studies of acetate, the most abundantly circulating SCFA in humans [157]. Several animal [152, 158–164] and some human [165–168] studies have shown beneficial effects of increased dietary sources of acetate and corresponding stimulation of microbial acetate production. These benefits include weight homeostasis influenced by satiety and appetite control [158, 160], resistance to weight gain and adiposity in the presence of a high fat diet [152, 163], and improving glucose regulation and insulin sensitivity [163]. However, recent rodent trials have also demonstrated opposite effects, finding that increased acetate turnover, resulting in part from gut microbiota acetate production, can contribute to obesity via weight gain and insulin resistance [169–171]. The role of sex differences in these processes is not established, but these discrepant findings point to the complexity and uncertainty of the role of microbiota-produced SCFAs in obesity development that needs to be considered when evaluating the role of the gut microbiome in obesity and CVD risk factors more generally.

Although evidence is mounting for the microbiota as a mediator of diet on obesity and other metabolic diseases [143], additional longitudinal research in humans is needed to elucidate the complex interplay and directionality of the microbiota-obesity relationship as well as to understand the influence of and resultant sex differences in these processes.

## Future directions and conclusion

Recent investigations have highlighted key sex differences with respect to CVD prevalence, risk, and progression that may be driven by traditional risk factors including dyslipidemia, hypertension, insulin resistance, and obesity. Furthermore, while preliminary research has implicated a potential role of the microbiome in mediating the relationships of upstream risk factors and CVD, sexual dimorphism in this research area remains largely unexplored. Future studies must clearly isolate the role of sex from diet, host health, age, ethnicity, and environment to conclusively identify potential biological sex differences in the gut microbiome. In particular, prospective study designs are necessary to document temporal changes in the gut microbiome as they relate to physiological hormonal cycles and critical hormonal time periods that associate with determinants of CVD, including puberty and menopause. Initial studies suggest that microbiome-associated toll-like receptor signaling cascades, bile acid metabolism, and steroid hormone modulation may be important drivers in sex differences in CVD risk. Additional mechanistic studies are necessary to discover how gut microbiota may initiate or mediate key sex-specific biological determinants of CVD, particularly through the serum metabolome, in the general population. Future evidence derived from mechanistic studies may pave the way for potential low risk interventions involving microbiota to reduce CVD risk throughout the lifespan.

## Data Availability

All data analyzed during this review article are included in this published manuscript in-text or cited with a reference to the data source.
